# Analysis of two-phase air-water annular flow in U-bends

**DOI:** 10.1016/j.heliyon.2020.e05818

**Published:** 2020-12-28

**Authors:** J. López, N. Ratkovich, E. Pereyra

**Affiliations:** a McDougall School of Petroleum Engineering, The University of Tulsa, Tulsa, USA; b Chemical Engineering Department, Universidad de Los Andes, Bogotá, Colombia

**Keywords:** Annular two-phase flow, Computational fluid dynamics (CFD), Volume of fluid (VOF), Superficial gas velocity, Curvature radius

## Abstract

This paper presents an experimental and numerical study of gas-liquid annular flow in horizontal 180 U-bends. The paper aims to study the effect of bend curvature radius and superficial gas velocity in the liquid film's behavior and annular flow characteristics. The study is divided into three sections. The first section corresponds to the experimental methodology and results. The second section compresses the validation of the computational fluid dynamic (CFD) model with the experimental results. Finally, the last section presents the CFD estimation of additional variables that cannot be acquired with the existing experimental setup. The experimental results provide an initial understanding of the multiphase mixture obtained using optical techniques (i.e., High-Speed Filming (HSF) analysis). The comparison between the experiments and the numerical simulations is presented, and a reasonable agreement is observed between both approaches. Finally, additional results such as film distribution and rotation before and after the bend are extracted from the CFD simulations.

## 1 Introduction

Two-phase gas-liquid flows in pipelines are commonly present in several industries as Oil and Gas, nuclear, and chemical. Return bends or U-bends are found in several applications, and their unique configuration affects the behavior of the multiphase mixture. Gravitational, interfacial, and centrifugal forces affect the gas-liquid mixture's behavior as it passes through the bend. The bend's effect on the flow depends on the flow pattern, phase velocities, flow direction (i.e., upward, downward, horizontal), and the bend configuration (i.e., vertical, horizontal, inclined, and curvature radius).

Parameters as liquid film distribution, pressure loss, wall shear stress, and liquid holdup are critical parameters in the study of gas-liquid annular flow. Forcing annular flow through a 180 bend may lead to operational issues such as secondary flow, flow separation, pressure pulsation, and pipe drying. In turn, these can lead to pipeline integrity problems such as burnout, corrosion, or tube failure.

In horizontal pipes, gravity causes asymmetry in the flow pattern distribution, especially in annular flow, where the liquid film distribution accumulates at the bottom of the tube. This asymmetry gets more relevant where this flow pattern occurs in a bend due to centrifugal forces. Therefore, this asymmetry and curvature's influence are essential to describe the annular flow pattern [1] correctly. There are four mechanisms present in annular flow: (1) dispersion of liquid drops in the gas core by surface tension force, (2) dispersion of liquid film by secondary gas flow, (3) dispersion of the film by the liquid waves, and (4) liquid drop entrainment in the gas core [2, 3]. The effect of the bend on these mechanisms is of particular interest [4].

An extensive review of the available literature on the subject reveals that, despite the extensive research conducted on annular flow through U-bends, they do not consider the combined effect of superficial gas velocity and bend curvature radius. Especially in the effect that it may have in the liquid film distribution up and downstream of the bend. Studies as Abdulkadir et al. [1], Bandyopadhyay et al. [5], or Ribeiro et al. [6], among others, studied annular flow in bends from an experimental approximation. The studies focus mainly on developing the separator's flow downstream, the pressure drop through the bend, and analysis of liquid entrainment in the gas core. Some other studies as Abdulkadir [7], or Ghosh et al. [8], performed numerical studies, where parameters as erosion due to solid transport, pressure drop, or liquid velocity profiles were analyzed and studied.

A summary of some of the relevant papers reviewed involving annular U-bends conditions is presented in Table 1. Studies comparing experimental and numerical simulations in annular two-phase flow in U-bends are limited, as observed in the table. Furthermore, only a handful explore the effect that the curvature has on the configuration of annular flow.

**Table 1:** Related literature on annular flow in bends.

Author	Type of study	Curvature Radius (R/D)	Superficial Gas Velocity [m/s]	Measured Parameters
Abdulkadir [7]	CFD	3	3.5–16.1	Film thickness Film fraction Liquid Holdup
Abdulkadir, et al. [1]	Experimental	3	3.5–16.1	Mean film fraction
Azzi, et al. [9]	Mechanistic model	-	-	Pressure behavior in bend
Bandyopadhyay, et al. [5]	Experimental and CFD	3.4–10.5	0.2–1.9	Pressure drop
Ribeiro, et al. [6]	Experimental	5	14–25	Drop size of entrained liquid
Chen, et al. [10]	Experimental	1.9	0.2–2.1	Pressure drop
Da Silva Lima and Thome [11]	Experimental	1.9–8.3	NA	Heat transfer Pressure drop
Ghosh, et al. [8]	CFD	8.3	Oil-water mixture	Phase distribution Phase velocity profile Liquid holdup
de Oliveira, et al. [12]	Experimental	6.1–12.2	0.4–20	Pressure drop Liquid holdup

Despite the comprehensive studies in gas-liquid annular flow in bends, presented in the table above, there are no studies where the bend's effect in the liquid film is studied. Therefore, this work aims to study a horizontal U-bend's effect in the liquid film's behavior in annular gas-liquids two-phase flow. Specifically, it analyzes the effect of bend curvature radius and gas velocity in the liquid film's behavior before, after, and at the bend. The experimental results provide an initial approximation and serve as a validation technique for the CFD model. Then, once validated, additional variables of interest are extracted and analyzed from the CFD simulations.

## 2 Methodology

This section describes the experimental and numerical methodology followed in this study.

### 2.1 Experimental setup

**Figure 1:**
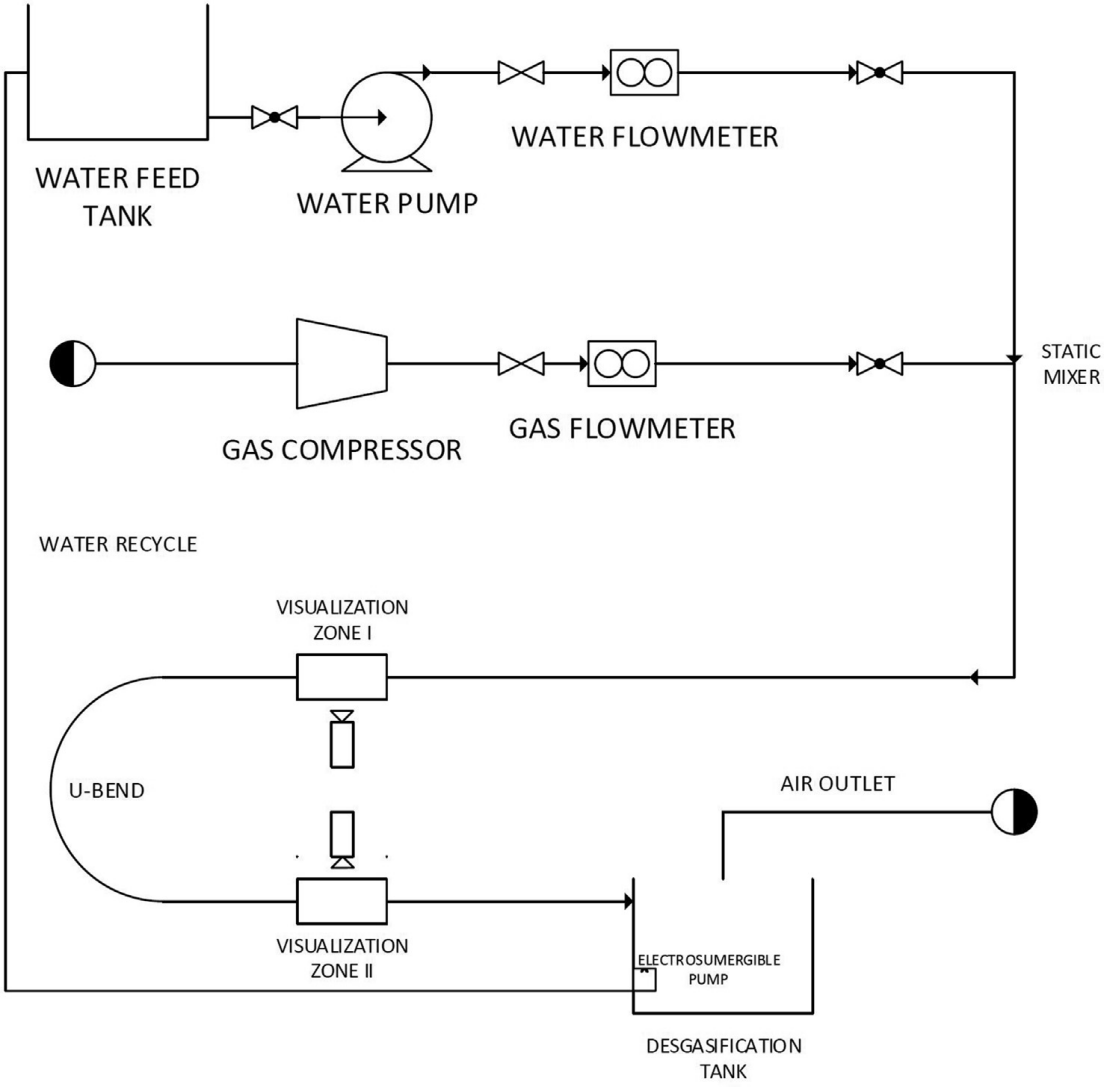
Universidad de Los Andes low-pressure flow in bends facility schematic.

The *Universidad de Los Andes* low-pressure flow in bends facility was designed, constructed, and used in this study. The facility is divided into three sections: fluids delivery system, test section, and return and separations section. A schematic of the facility is shown in Figure 1. The measurements were performed using optical techniques. The facility comprises four zones: intake, mixing, test section, and separation and return (Figure 1).

City of Bogotá tap water (density and viscosity of 997.56 kg·m^−3^ and 8.88e^−4^ kg·m^−1^·s^−1^, respectively) and compressed filter air (density of 1.18 kg·m^−3^ and viscosity of 1.8e-5 kg·m^−1^·s^−1^)were used as testing fluids. The fluids delivery system corresponds to the preparation and metering of the water and air. A 690-kPa (100-psi) air supply line was used for the air. The flow is filtered to avoid solid particles or humidity in the stream. The air supply can reach flows of up to $1.66\times 10^{-3}-m^{3}/s$ (100-LPM), with a precision of $8.33\times 10^{-6}-m^{3}/s$ (0.5-SLPM). The air is metered and controlled using a standard Dwyer® rotameter. Following the flow meter, a check valve prevents the reverse flow of the liquid into the rotameter. A centrifugal 0.5-HP pump was used to flow the water from a $0.2-m^{3}$ storage tank into the mixing and test section. For safety reasons, a check valve was located between the storage tank and the pump. Once the fluid exits the pump, it passes through a gate valve, allowing manual control of the flow, and then sent to a Hedland® flowmeter where the maximum flow rate that can be achieved is $2.5\times 10^{-4}-m^{3}/s$ (15-LPM), with a precision of $8.33\times 10^{-6}\;m^{3}/s$ (0.5-SLPM). The mixing of the fluids is achieved by a static T-shape mixer, which generates the multiphase mixture and diverts it to the test section.

The test section is divided into three parts: developing region, bend, and return region. The developing region consists of a straight horizontal 2-m long pipe made of transparent polymethylmethacrylate with a 14-mm inner diameter. An observation zone is located at 1.7-m from the inlet. This observation zone consists of a polymethyl methacrylate box that surrounds the pipe full of water to avoid light diffraction when the high-speed videos are recorded. Additionally, a high intensity LED light is placed in the background to provide enough illumination in the zone. The high-speed videos are obtained in the visualization zone. The dimensions of the developing region ensure the development of the flow as required [13]. The bend section consists of a 14-mm inner diameter glass U-bend, attached to the developing and return regions by polypropylene couplings (these couplings allow the attachment of different bend configurations). The return section is an additional 1.4-m polymethyl methacrylate pipe after the U-bend, also fitted with an observation box. The second box is located at 0.45-m from the bend. The return pipe takes the multiphase mixture to the last section of the facility (i.e., the separation and liquid return). A degasification atmospheric tank is used to separate the gas from the liquid and recirculate the water to the feed tank using an 0.25-HP electro-submersible pump that connects both tanks.

**Figure 2:**
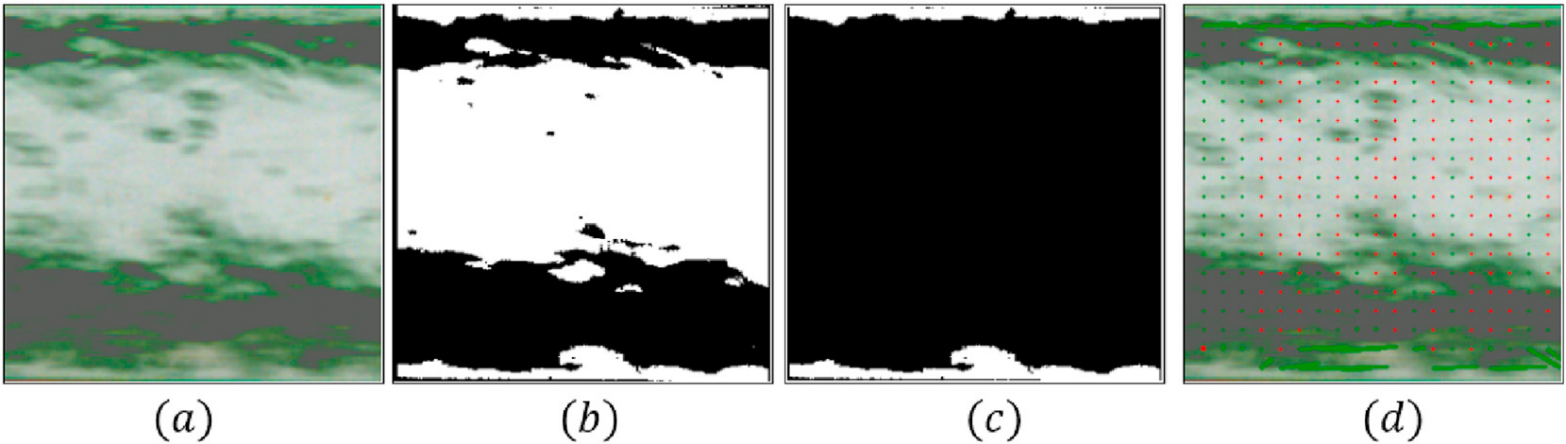
High Speed Filming analysis process. (a) raw frame extracted from the high speed video. (b) Black and White frame. (c) Fillage of shapes, identify liquid film and entrain droplets. (d) Centroid of interface tracking cells.

As previously mentioned, different U-bends geometries can be attached to the test loop. For this study, three curvature radii of the bend were selected. The R/D relationships (curvature radius – pipe diameter) of the chosen curvature were: 2, 3.5, and 5. The facility allows identifying the flow pattern and quantifying the holdup in a straight section of the pipe (i.e., before and after the bend). This is performed by optical methods, namely, high-speed filming (HSF) analysis. A Photron® FASTCAM 100KC camera was used with a Vivitar® 28-210 macro lens to record videos of up to 10900 frames per second. The videos were collected at 2000 frames per second, and a 1.5 s physical time was captured using a video with 3000 frames. These videos were processed and analyzed using the methodology proposed by López, et al. [14], where a four-step process is performed. The videos are trimmed to obtain square footage, in which only the pipe is captured (the remainder of the visualization box must be trimmed). This process is needed to perform the analysis just of the two-phase mixture and avoid using computational resources analyzing other sections of the visualization box that do not correspond to the multiphase mix (i.e., pipe walls and outside the pipe). Nevertheless, a maximum resolution of the image is required to differentiate the phases and track the interphase; thus, it is crucial to reduce image resolution in the trimming process. The second step is to apply a threshold technique in the video. This step converts each frame into black and white. This method allows the differentiation of the bubbles' contours and tracks the interphase between the phases per frame. With the contours already identified, the next step is to fill the shapes. This process fills the white water shapes and leaves the gas shapes in black, generating a final image in black and white. A graphical description of the four steps is presented in Figure 2. For a further description of the process, refer to López, et al. [14].

**Figure 3:**
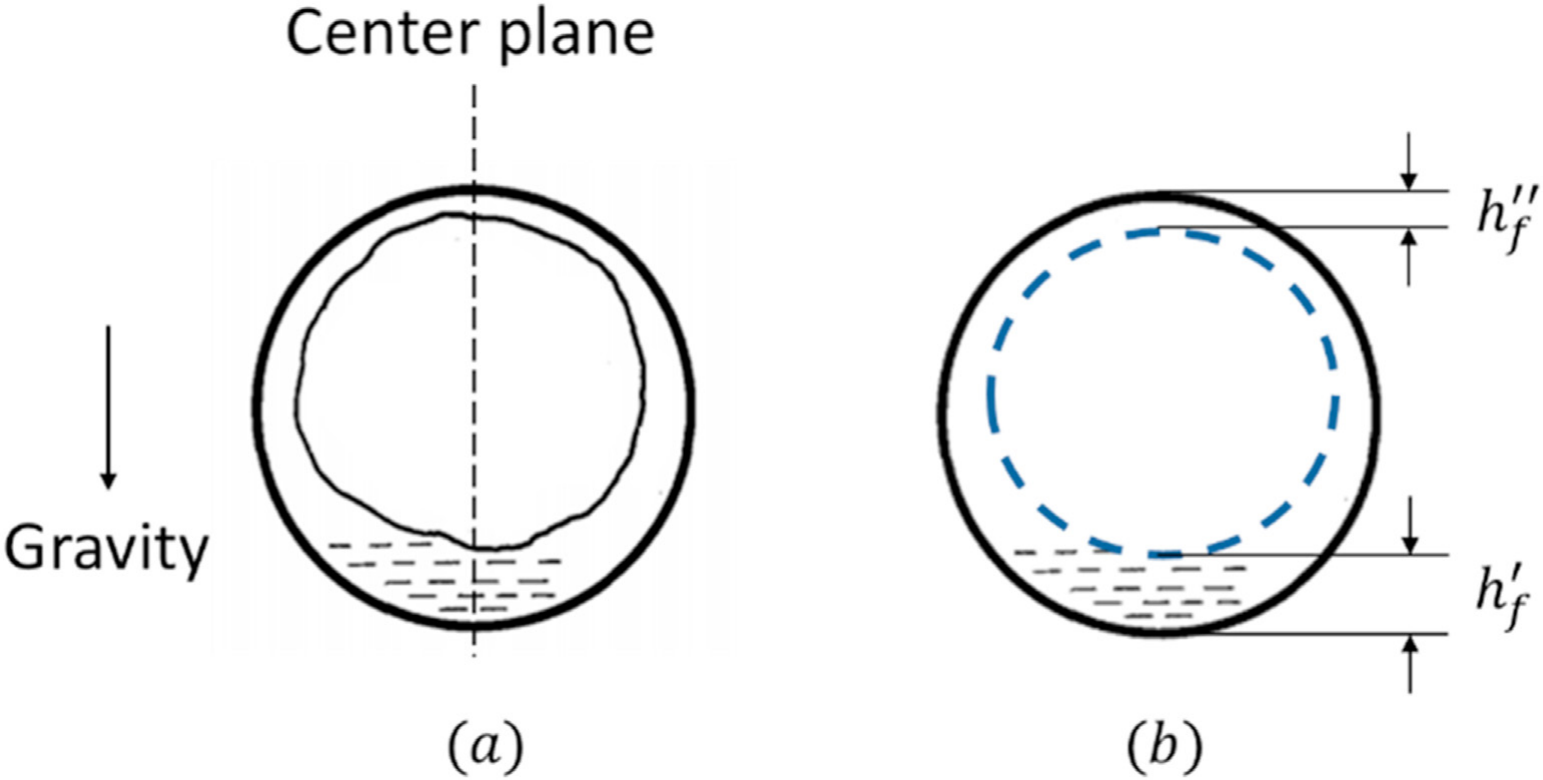
Liquid holdup approximation based on film height. (a) A cross-sectional view of liquid film distribution in annular flow. (b) Double circle approximation.

The plane where the videos are recorded corresponds to the center plane of the pipeline. Therefore, the percentage of white pixels obtained during the video processing corresponds to the liquid film's height at the pipeline's center plane. To convert this value into liquid holdup, an approximation similar to the one proposed by Chen, et al. [15] was followed. The methodology is based on a double circle approximation to estimate the wetted wall fraction and liquid holdup. A schematic of the proposed approximation is presented in Figure 3.

The values for the height of the lower liquid film ($h_{f}^{\text{′}}$) and the upper section of the film ($h_{f}^{\text{″}}$) are obtained from the video analysis in pixels, which then are converted to meters. Then, the liquid holdup ($H_{L}$) can be calculated straightforwardly from geometrical relationships as:

(1) $$H_{L}=\frac{\left(2d_{p}\left(h_{f}^{\prime }+h_{f}^{\prime \prime }\right)-{\left(h_{f}^{\prime }-h_{f}^{\prime \prime }\right)}^{2}\right)}{d_{p}^{2}}$$

Table 2 presents the experiments matrix tested. A single superficial liquid velocity and two superficial gas velocities were tested. This combination was tested for three different curvature ratios. The combination of studied parameters allows the performance of sensitivity analysis in the phenomenon and allows the determination of the bend's effect in the annular flow. It also presents the terminology of each case, which is used in the presentation of the results.

**Table 2:** Description of the studied cases.

$v_{\mathrm{sg}}\;\left[m/s\right]$	$v_{\mathrm{SL}}\;\left[m/s\right]$	R/D^1^
9.74	1.08	2
11.04		
9.74		3.5
11.04		
9.74		5
11.04		

1 Curvature Radius vs. pipe inner diameter relationship.

### 2.2 Numerical model

**Figure 4:**
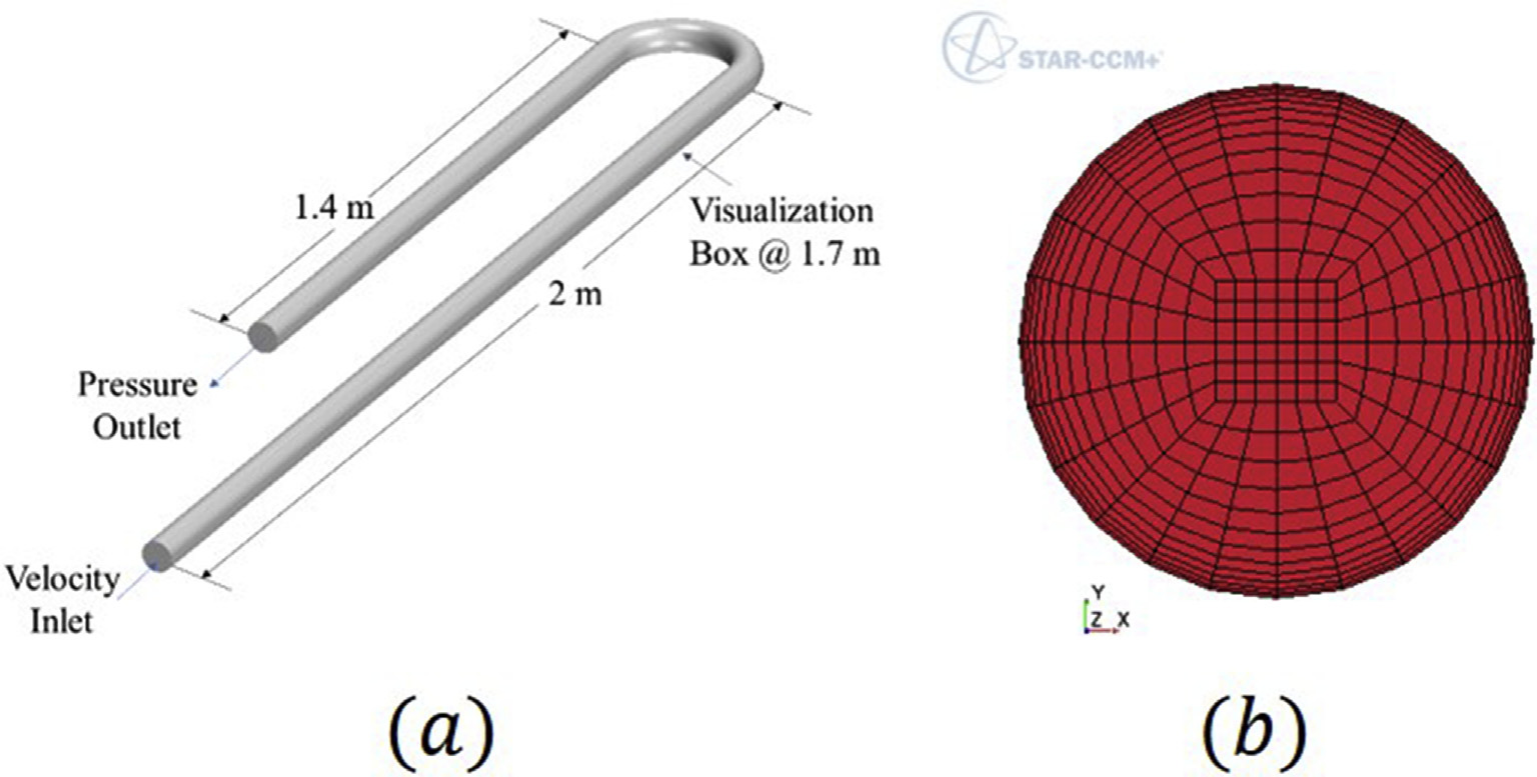
Simulation pre-processing. (a) Facility CAD and dimension. (b) Mesh distribution in a transversal section of the pipe.

STAR-CCM+® v13.04 (Siemens) was used for the computational fluid dynamics simulations, following the López, et al. [15], Tkaczyk and Morvan [16], and Pineda-Pérez, et al. [17] procedures. Within the simulations, three main steps were followed: pre-processing, processing, and post-processing. Pre-processing consists of defining the geometry, mesh, physics, boundary conditions, and initial conditions for each case. The Computer-Aided Design (CAD) modeler available in STAR-CCM+ was used to create a facility's physical description. Figure 4a shows the dimensions of the geometry simulated in the facility. The same dimensions that the experimental facility was simulated in the simulations. This was to ensure that the two-phase mixture was fully developed before the bend and the visualization box. A structured mesh was performed, based on the CAD model, as shown in Figure 4b.

This mesh allows a constant cell number in the inlet, outlet, and transversal section of the pipe, resulting in a constant density of cells in the entire domain, and therefore, direct control over cell size and distribution [18]. This orthogonal mesh type has been recommended for the simulation of two-phase flow in pipes [19]. Since this study focuses on annular flow behavior, a refinement near the wall was carried out to correctly solve the boundary layer, and a proper wall treatment by the turbulence model [20]. The refinement was achieved by applying a hyperbolic progression from the wall into the pipe's core, as seen in the previous figure. Finally, the transversal mesh's extrusion is performed in a longitudinal direction to complete the meshing process. It is worth mentioning that the complete test section of the experimental facility was modeled in the software CAD and correspondingly meshed.

As for the used physics models, the Volume of Fluid (VOF) multiphase model with a High-Resolution Interface Capturing (HRIC) method was selected to simulate the liquid-gas mixture. This model allows the gas-liquid interface to be tracked using the same transport model for each phase and includes an additional transport equation for each phase's volume fraction. The mixtures' properties are calculated by each phase's contribution, taking into account each volume fraction [7, 20]. The HRIC method is designed to mimic the convective transport of immiscible fluid (e.g., water and air), resulting in a suitable scheme for tracking sharp interfaces in significant spatial variations of phase volume fractions. The VOF with the HRIC method uses a second-order downwind scheme that accurately captures interfaces that are perpendicular to the flow direction. However, if the two-phase interface is parallel to the flow direction(as in this case), the HRIC scheme tends to wrinkle it and aligns it with the mesh lines [21]. This promotes convergence by having the mesh cells full of only one phase and the mesh boundary interface. To avoid the misalignment of the interface, a correction can be made to the scheme. This correction is called the interface angle factor. This angle correction is based on the angle formed between the interface and the cell-face surface vector. If the angle factor tends to zero, no correction is performed. Since the free surface is not smooth, and not following the grid lines, a larger value of 0.2 was used [22]. The gas-phase was selected as the primary phase and the liquid as the second phase; this is necessary to compute the phase interaction driven by tension forces.

A parameter of importance in the VOF modeling is the sharpening factor, which reduces the numerical diffusion in the domain by adding a term in the transport equation [18, 22]. This factor varies from 0.0 to 1.0, where 0.0 means no reduction of the numerical diffusion. Although the 0.0 value could obtain excellent results using the standard discretization scheme, a sharper interface is obtained with higher values of the sharpening factor. Pineda-Pérez, et al. [17] commented that a sharpening factor of 1 allows a better definition of the flow patterns since there is a better definition of the disperse phase (the liquid film). Additionally, since an annular flow experiences a sharp interface between the gas core and the liquid film, a value of 1 was selected for the simulations. A segregated model was applied to solve the flow equations, using the second-order upwind convection scheme. Since the two-phase phenomenon is time-dependent, an implicit unsteady study was performed. The liquid was modeled with constant densities, and the gas was model as a real gas. The remaining fluid properties (e.g., viscosities and surface tension) corresponded to the experimental values. Also, a gravity component on the momentum equation was included.

The κ-ω SST (shear stress transport model) was selected to model the turbulence in the system. This model is a two-equation model that solves the turbulent kinetic energy and its dissipation. One of the reported advantages of the κ-ω model over other turbulence models is its improved performance for boundary layers under adverse pressure gradients. However, possibly the most significant advantage is that it may be applied throughout the boundary layer, including the viscous-dominated region, without further modification [23]. Nevertheless, the model's disadvantage is that boundary layer computations are sensitive to the free stream's values. This means an extreme sensitivity to inlet boundary conditions for internal flows. Simulating the complete developing region pipe and the Shear Stress Transport correction can address this issue. The transformed equation looks similar to the standard model but adds a non-conservative cross-diffusion term. This other term potentially makes this model give identical results to the κ-ε model. Then, Menter [24] suggested using a blending function that would include the cross-diffusion term far from walls (the κ- ε model in the far-field), but not near the wall (κ-ω in the boundary layer), and thus, take advantage of both methods were they perform the best.

**Figure 5:**
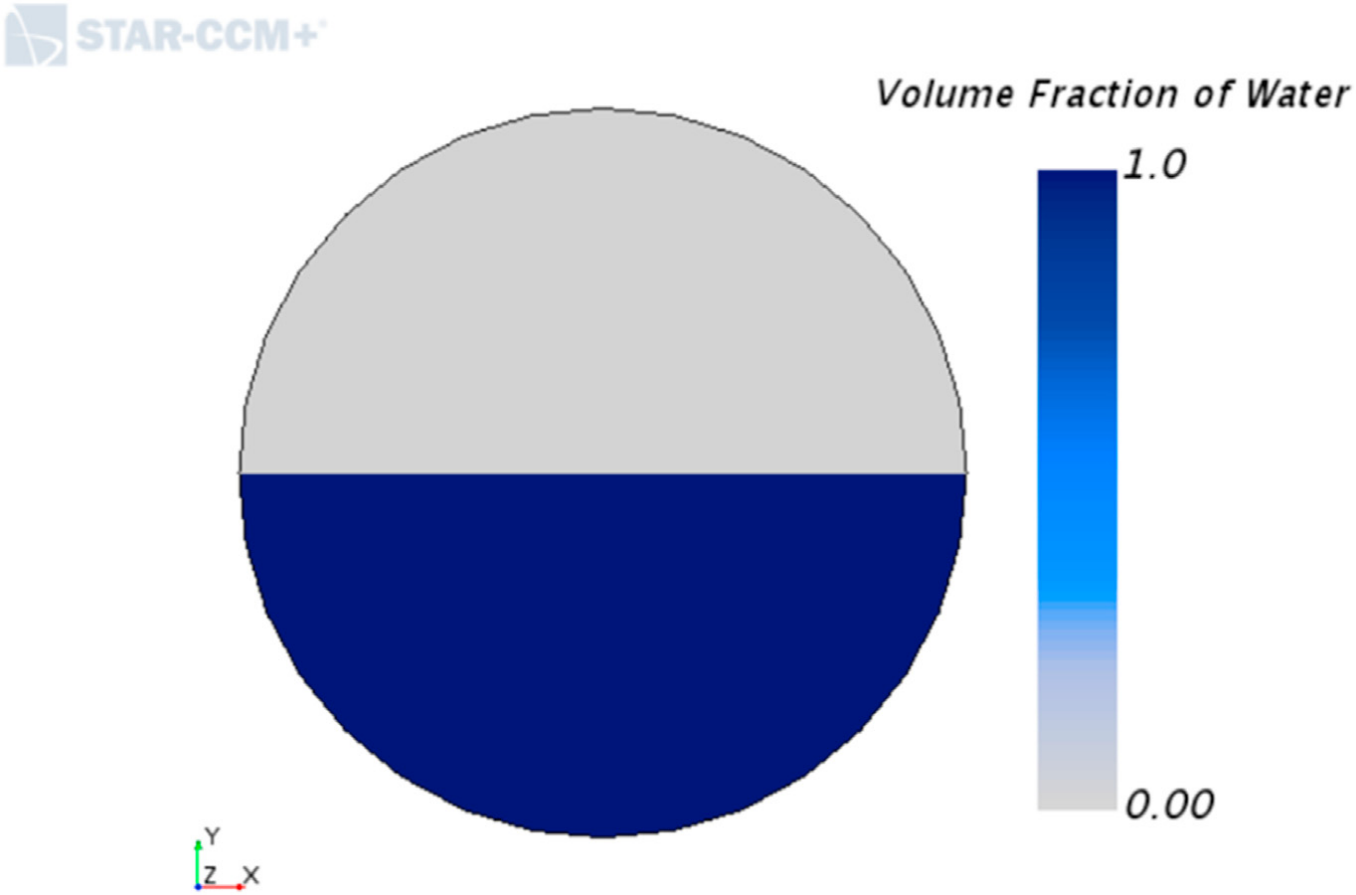
Volume fraction boundary condition at the inlet.

The inlet of the phases was set up as a velocity inlet where the phases are segregated, as observed in Figure 5. The phase velocities are set up accordingly to match the volumetric flowrates of each experimental condition. This was performed to match the experimental conditions. Since the whole developing region was simulated, enough pipe length is available to ensure flow development. The outlet boundary was modeled as a pressure outlet, emulating the degasification tank in the facilities.

The Convective Courant Number was used to calculate the time step of each of the simulations. It is defined as:

(2) $$C=\frac{u\Delta t}{\Delta x}$$

This parameter determines the simulation time step (Δt) based on the cell size of the mesh (Δx) and the phase velocities (u). If the Courant number is 1, a particle of fluid will move one cell per time step. If larger, in a one-time step, the fluid will move more than one cell. Therefore, an assumption of values must be performed in the intermediate cells. Consequently, a Courant Number inferior to 1 is required to model the phenomenon correctly. It is suggested to use a low CFL number when the HRIC model is used (e.g., below 0.35) [18]. This is to allow enough time for the interface within each grid to be adequately solved. Thus, the maximum value of the CFL for all the simulations was fixed at 0.25. The time step for each simulation was calculated using the double of the maximum velocity between the phases. Since the gas velocity was always higher than the liquid velocity, the time step was calculated using the double of the superficial gas velocity and the axial space between cells of the mesh (2-mm). The resulting time step varied between 1.53 μs and 2.29 μs. Finally, five iterations per time step were defined.

The processing phase of the model is the fundamental computational part of the methodology. The previously selected equations are solved using the finite volumes method. The initial condition for each simulation is a pipe is full of non-moving air; this is because the primary phase is the air, and therefore, the computational cost of the initial steps focuses on the pipe inlet. The stopping criterion is when it takes the slow fluid (water) to pass through the bend twice. This provided flow development and avoided divergence errors.

Finally, the post-processing is the procedure in which the desired results are extracted for each simulation. For this study, variables as flow pattern, the liquid holdup in different locations, liquid film distribution in the bend, and velocity profiles were obtained.

## 3 Results and discussion

This section presents and discusses the experimental and CFD results. First, the experimental results are presented. Next, the CFD results are presented where a grid independence study is initially performed, and then the validation of the CFD results with the experimental ones is presented. Finally, additional CFD results are presented. These results cannot be obtained with the current experimental setup.

### 3.1 Experimental results and discussion

Table 3 presents the liquid holdup results obtained by the video analysis methodology. The average holdup and the standard deviation of the trend in time are reported.

**Table 3:** Liquid holdup results.

Case	$v_{\mathrm{sg}}\;\left[m/s\right]$	$v_{\mathrm{SL}}\;\left[m/s\right]$	R/D	$H_{L}$ [-]	$\sigma \;H_{L}\;\left[-\right]$
1	9.74	1.08	2	0.3026	0.0741
2	11.04	1.08	2	0.2768	0.0839
3	9.74	1.08	3.5	0.2470	0.0602
4	11.04	1.08	3.5	0.2302	0.0624
5	9.74	1.08	5	0.2101	0.0654
6	11.04	1.08	5	0.2022	0.0637

An impressive result extracted from the experimental results is the effect of the bend in the liquid holdup. As the curvature radio increases, so does the liquid holdup. This reduction in the mean value suggests that the gas travels at high velocities at a lower curvature radius. Therefore, the gas goes through the bend faster than the liquid and causes an accumulation of the liquid before the bend. This leads to higher liquid hold up. As the curvature increases, the liquid holdup decreases as the liquid passes through the bend more rapidly and with more ease.

**Figure 6:**
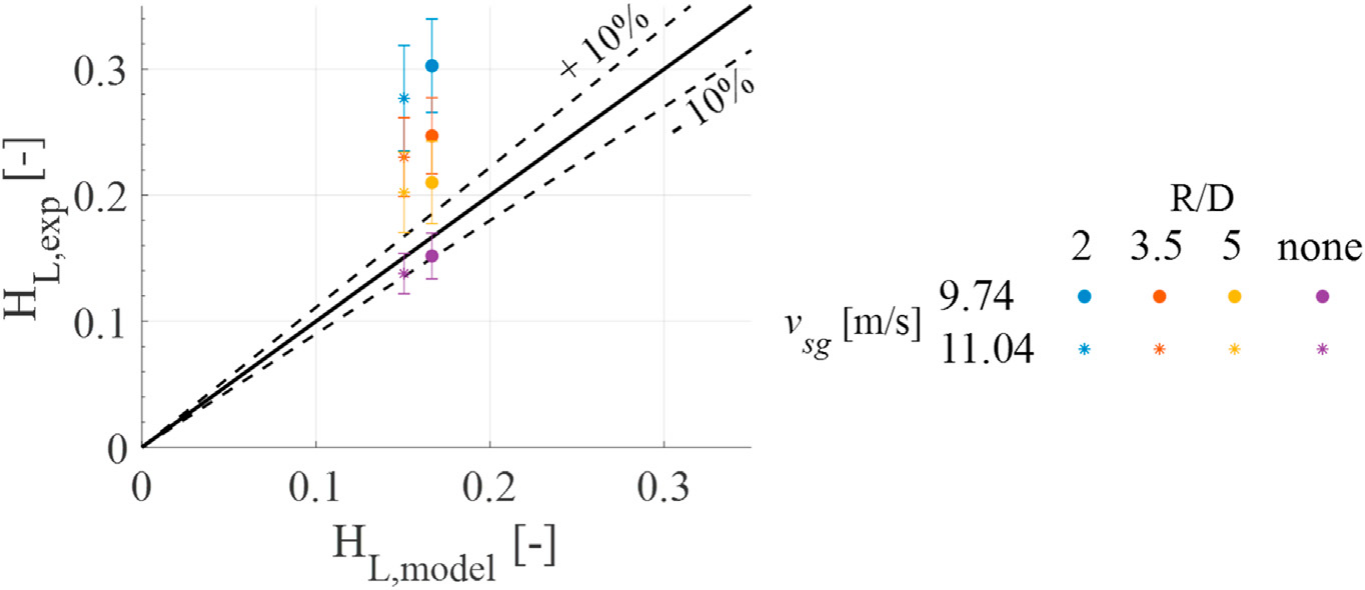
Liquid holdup comparison against Zhang, et al. [25] unified model.

These results can also be compared with the prediction of annular flow in fully developed pipe flow. Two additional experimental points were generated where no bend was included in the experimental setup. This means that the facility test section's bend and return region was removed, and the two-phase mixture was diverted directly into the degasification tank and liquid return. This was performed to obtain experimental cases without a bend and validate the measuring technique (i.e., HSF analysis) with available gas-liquid annular flow models. The validation is performed with Zhang, et al. [25] unified model for gas-liquid mixture in pipelines. The model is based on the hydrodynamics of slug flow and was developed to predict flow pattern transitions, pressure gradient, liquid holdup, and slug characteristics in gas-liquid pipe flow at all inclination angles from −90° to 90° from horizontal. Since the model has been widely validated in the literature, it is used to validate the developed measuring technique and calculation approach. The comparison between the experimental results and Zhang, et al. [25] model predictions are presented in Figure 6.

An initial observation of the figure concludes that the experimental data disagrees with the Zhang et al. model's predictions. This discrepancy is attributed to the inclusion of the bend. The model slightly underpredicts the liquid holdup for the experimental cases with no bend (showed as purple markers). However, the measured holdup lies within a 10% error of the predicted value. This means that the presented experimental technique correctly predicts the behavior of liquid holdup in pies. Once the bend is included in the facility, the experimental measurement seems to overpredict the liquid holdup. This difference is attributed to the effect that the bend has on the flow upstream of it. It is worth mentioning that holdup's value is measured upstream of the bend in the developing region regarding the curvature tested. Then, the increase in the measured liquid hold up means an accumulation of liquid upstream of the bend. It can also be observed that the smaller the bend, the higher is the value of holdup, meaning the higher the accumulation of liquid before the bend.

**Figure 7:**
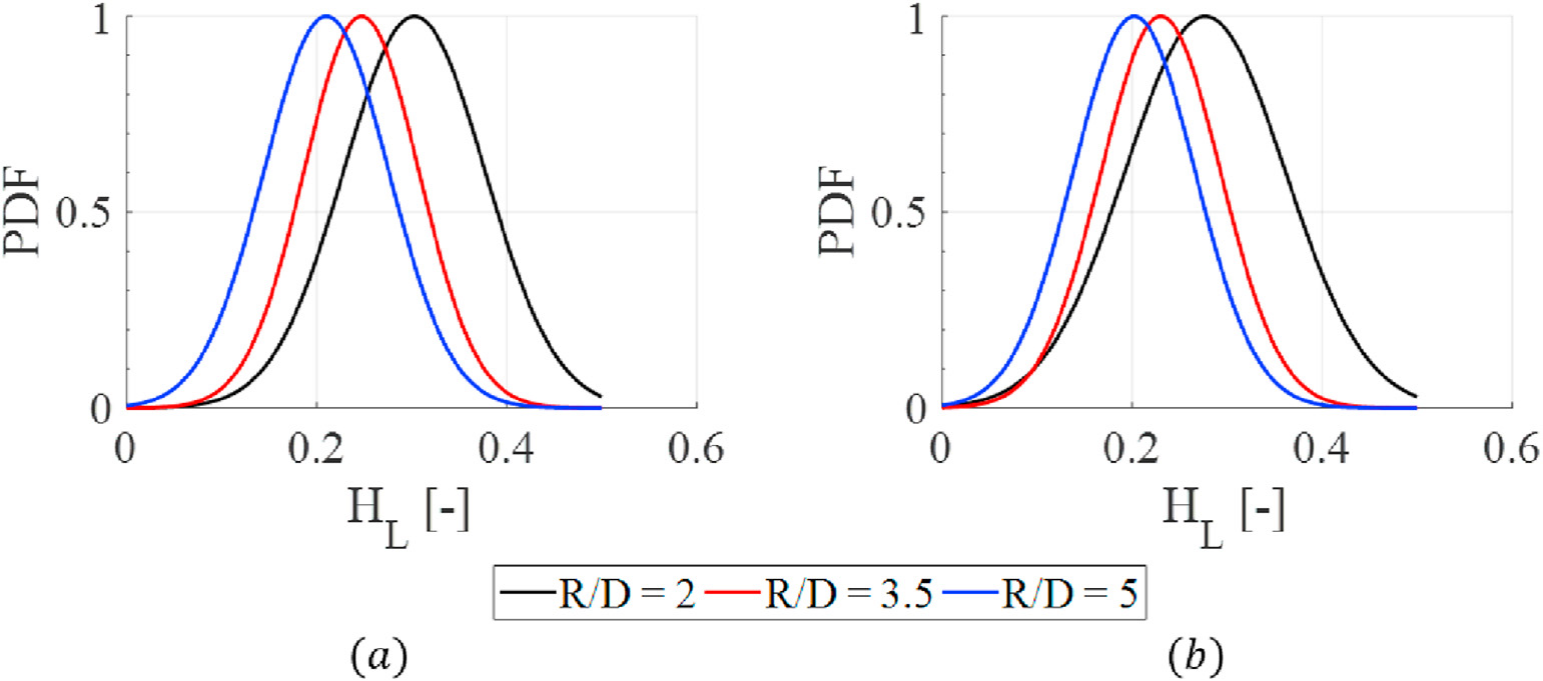
Experimental results. (a) $v_{sg}=9.74\;m/s$. (b) $v_{sg}=11.04\;m/s$.

The experimental results are also presented as normalized Probability Density Function (PDF). Observing the liquid holdup results in a PDF removes the noise of observing the results a time trend. In this way, an explicit comparison of the liquid holdup's dynamic behavior can be obtained between the cases. The PDF's are presented in Figure 7.

The bend and gas flow rate effect can also be explained by analyzing centrifugal forces in the bend. At a tighter curvature radius, a higher liquid accumulation before the bend was observed. Therefore, more gas mass flux is in the bend. This led the gas to experience higher centrifugal forces. This is results accelerates the gas through the bend, causing an accumulation of liquid before the same. As the curvature increases, the effect of centrifugal forces in the gas phase; thus, less accumulation of the liquid film is observed. A modified gas Froude number that relates the relationship between inertial and centrifugal forces [7] can be applied to quantify the effect of the bend, as:

(3) $$Fr_{g}=\frac{v_{sL}^{2}\rho_{L}}{\left(\rho_{L}-\rho_{g}\right)Rg}\;\left(1-\frac{\rho_{g}v_{sg}^{2}}{\rho_{L}v_{sL}^{2}}\right)$$

When $Fr_{g}>1,$ the centrifugal force of the gas phase is greater than the inertial force. In other words, the gas phase will go faster through the bend, and therefore a higher liquid accumulation before the bend will be observed. Table 4 presents the results of the modified Froude number for the studied cases.

**Table 4:** Modified gas Froude number.

Case	$v_{\mathrm{sg}}\;\left[m/s\right]$	$v_{\mathrm{SL}}\;\left[m/s\right]$	R/D	$Fr_{g}$
1	9.74	1.08	2	3.8633
2	11.04	1.08	2	3.7528
3	9.74	1.08	3.5	2.2076
4	11.04	1.08	3.5	2.1445
5	9.74	1.08	5	1.5453
6	11.04	1.08	5	1.5011

The modified gas Froude number confirms that the tighter the bend, the higher the centrifugal force observed by the gas. This force balance can also be used to analyze the distribution of the liquid film in the bend. When $Fr_{g}>1$ the gas phase will be inside the bend since the centrifugal force is higher than the inertial one. The fast-moving gas will push the liquid film to the outside of the bend. When $Fr_{g}<1,$ the gas's inertial force is greater than the centrifugal force; hence, the gas will attempt to stay in its current trajectory and thus remains on the outside of the bend. The gas will push the liquid film into the inner section of the bend. Finally, if $Fr_{g}=1,$ a balance on the forces is presented in the system.

For a constant curvature radius, a higher superficial gas velocity will reduce the Froude number since the inertial force will increase. This is observed in the Froude number of the experimental results. This also means a critical superficial gas velocity for a curvature radius that will move the liquid film from the outside to the inside of the bend. Likewise, for a superficial gas velocity, a curvature radius will move the liquid film from the outside to the inside of the bend. Table 5 presents the critical superficial gas velocity for each of the tested bends. Due to facility limitations, the critical gas velocities were not able to be achieved. These conditions can be addressed in futures works.

**Table 5:** Critical gas velocity per curvature radius.

R/D	$v_{\mathrm{sg},c}\;\left[m/s\right]$
2	28.1994
3.5	24.7338
5	20.6959

Kalpakli [26] claimed that one of the U-bends' uses is as a flow conditioner, as it seems that the bend influences the mixture's behavior, especially in the behavior of the liquid film. These obtained results confirm the conclusions presented by the author.

### 3.2 Grid sensitivity study

**Figure 8:**
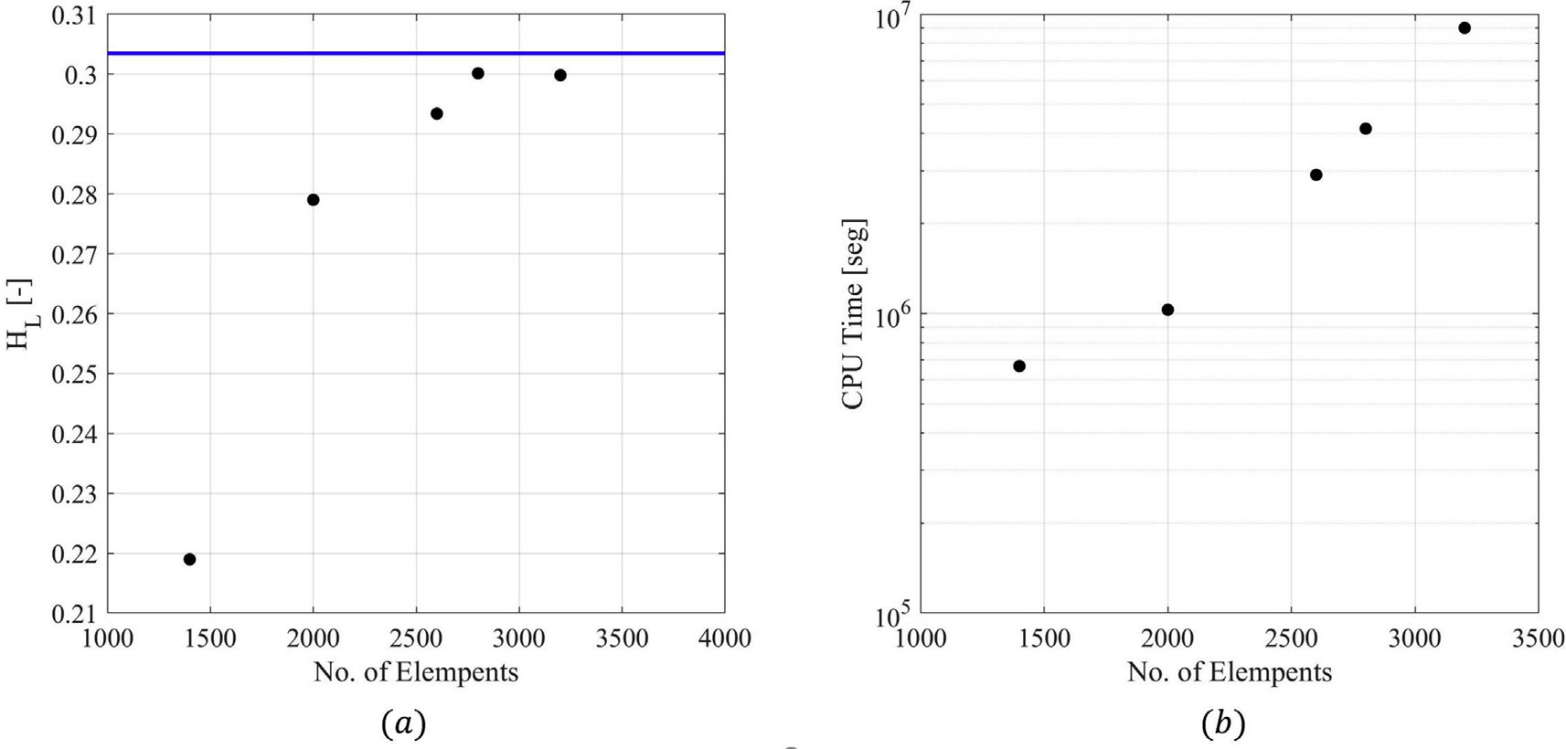
Grid independence test, changing in the longitudinal direction. (a) Holdup results vs number of elements. (b) CPU time vs number of elements.

To select the mesh size to be used in the CFD simulations, the effect of the discretization of the cross-sectional direction and the axial direction were studied in terms of the available experimental variables. Simulations were carried out, changing the number of divisions in both directions while maintaining the cell size ratio. Figure 8 shows the average liquid holdup, ${\text{H}}_{\text{L}}$ (average in the last 1.5 s of the simulation time) obtained by changing the number of divisions in the axial direction, maintaining a constant number of cells in the cross-sectional direction of 806. The figure also presents the simulation time of each evaluated mesh. Simulation times were calculated as the accumulated CPU Time divided by the number of processors used to run each simulation. This definition of time is used because the simulations were run in different CPUs with different nodes. It is observed that a higher number of divisions in the axial direction reduce the difference between CFD and experimental results (plotted as a continuous blue line). Two thousand six hundred (2600) divisions in the longitudinal section were selected to ensure tolerable error, provide a correct prediction of the phenomenon, and obtain a manageable computational time and resources.

**Figure 9:**
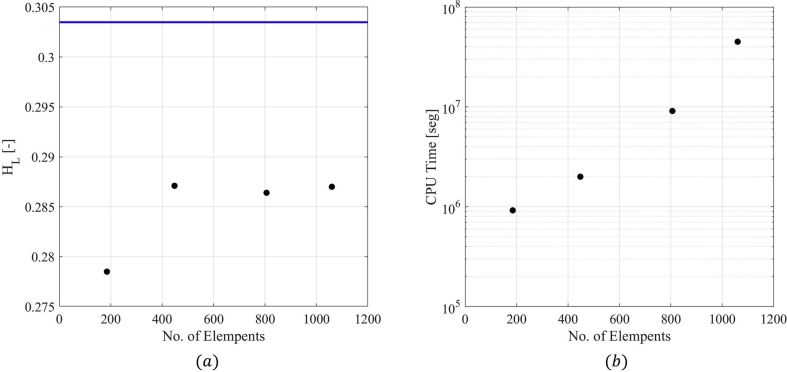
Grid independence test, changing in the cross-sectional direction. (a) Holdup results vs number of elements. (b) CPU time vs number of elements.

Once the number of elements in the longitudinal direction was chosen, four cross-section divisions were tested (i.e., 185, 448, 806, and 1060) to test the effect of the cross discretization. Figure 9 shows the cross-sectional divisions' impact on the average liquid holdup and the total simulation time. It can be observed in the figure that there is no clear trend in the results. However, a medium-size mesh (448) was selected since many divisions will produce a more considerable computational time.

### 3.3 CFD results and comparison

**Figure 10:**
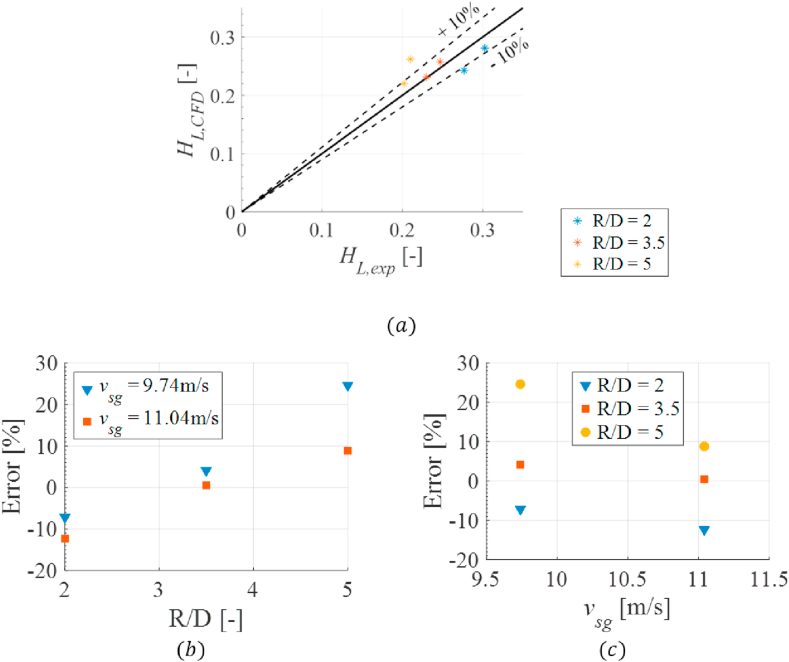
Experimental vs. CFD liquid holdup results. (a) Holdup comparison. (b) Error due to curvature radius. (c) Error due to superficial gas velocity.

The experimental results were compared with the simulations' outputs to validate the developed CFD model. The CDF data was post-processed to match the experimental output to perform a valid comparison. The liquid holdup value was obtained for all simulations in the pipe's cross-sectional plane in the coordinates where the visualization box is in the experimental facility. A value of liquid holdup is obtained every time step of the simulation, resulting in a time profile data set. The data corresponding to the last 1.5 s of the simulations was selected for the comparison. This was performed to ensure a flow development within the simulation and match the experiment's time recorded. Figure 10 and Table 6 presents the comparison between the experimental results and the CFD results. Figure 10a presents the comparison of the liquid holdup between the two techniques. Figure 10b presents the simulation error as a function of the curvature radius per every superficial gas velocity, and Figure 10c shows the simulation error as a function of the superficial gas velocity per curvature radius.

**Table 6:** Experimental vs. CFD liquid holdup error.

Case	$v_{\mathrm{sg}}\;\left[m/s\right]$	$v_{\mathrm{SL}}\;\left[m/s\right]$	R/D	$H_{L,\exp}$ [-]	$H_{L,\mathrm{CFD}}$ [-]	Error [%]
1	9.74	1.08	2	0.3026	0.2810	-7.142
2	11.04	1.08	2	0.2768	0.2427	-12.301
3	9.74	1.08	3.5	0.2470	0.2572	4.129
4	11.04	1.08	3.5	0.2302	0.2314	0.509
5	9.74	1.08	5	0.2101	0.2614	24.638
6	11.04	1.08	5	0.2022	0.2201	8.831

The model seems to underpredict the liquid holdup at tighter bends and overpredicts as the bend gets larger. This systematic error can be attributed to the HSF analysis, as the representation of the annular flow pattern from 3D to 2D representation, some elements are missing (i.e., dispersed droplets in the gas flow and a smooth interface between the liquid film and the gas flow). Also, it is observed that the error of the higher gas velocity is less than, the lower gas velocity. The presented comparison between the two techniques reveals that the CFD can accurately predict the average liquid holdup despite discrepancies in the results. Overall, the performance of the CFD code can be estimated by calculating the average percentage relative error $\left(\varepsilon_{1}\right)$, the average absolute percentage relative error $\left(\varepsilon_{2}\right)$, and the standard deviation of relative error $\left(\varepsilon_{3}\right)$, defined as:

(4) $$\varepsilon_{1}=\frac{1}{N}\overset{N}{\underset{i=1}{\sum }}e_{ir}$$

(5) $$\varepsilon_{2}=\frac{1}{N}\overset{N}{\underset{i=1}{\sum }}\left|e_{ir}\right|$$

(6) $$\varepsilon_{3}=\sqrt{\frac{\sum_{i=1}^{N}{\left(e_{ir}-\varepsilon_{1}\right)}^{2}}{N-1}}\;$$

where N is the number of simulations and $e_{ir}$ is the relative error. The overall performance of the CFD code is 3.11, 9.59, and 13.02 for $\varepsilon_{1}$, $\varepsilon_{2}$, and $\varepsilon_{3}$ respectively. The lower value of the percentage relative error and the absolute percentage relative error confirm that the CFD model correctly predicts the annular flow behavior. As with the model's overall performance, the error analysis can be performed to each variable changed in the conditions (i.e., superficial gas velocity and curvature radius). The results are presented in Table 7.

**Table 7:** CFD error for each studied variable.

R/D[−](all $v_{\mathrm{sg}}$)	$\varepsilon_{1}$ [%]	$\varepsilon_{2}$ [%]	$\varepsilon_{3}$ [%]
2	-9.7215	9.7215	18.5104
3.5	2.3190	2.3190	2.7939
5	16.7345	16.7345	22.2744
$v_{\mathrm{sg}}\;\left[m/s\right]$(all R/D)	$\varepsilon_{1}$ [%]	$\varepsilon_{2}$ [%]	$\varepsilon_{3}$ [%]
9.74	7.2083	11.9697	16.8757
11.04	-0.9870	7.2137	11.7688

**Figure 11:**
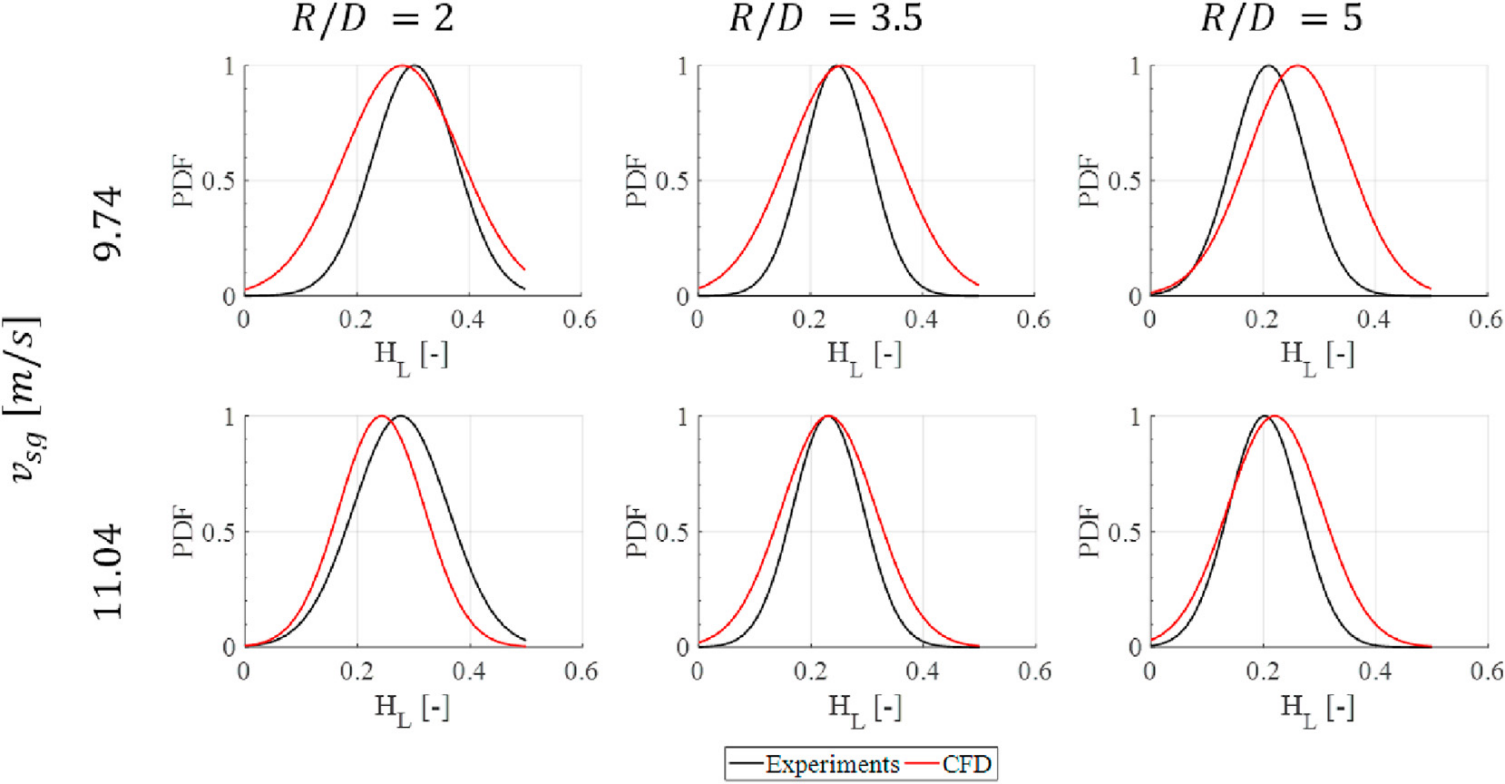
Experimental vs. CFD liquid holdup PDF results.

The error distribution evidence that the error in the prediction increases as the curvature radius increases (also observed in Figure 10a). The standard deviation of the error appears to be high for all cases due to the small number of experiments at each condition. Additionally, as with the experimental findings, the holdup results are shown as a PDF of the data. Figure 11 presents the comparison between the experimental and the numerical results.

In all cases, it can be observed that the CFD cases present a higher dispersion of the data, and therefore, the normal distributions seem broader than the experimental cases. This difference is due to the way the values of holdup are obtained in both techniques. In CFD, the liquid holdup was obtained in a cross-sectional plane of the pipe. Consequently, the phenomena' unsteady behavior is captured as it passes through that transversal section, meaning that an area approximation of the holdup is used. For the HSF cases, an analysis is performed in a volumetric section of the pipe. The volumetric approximation reduces the signal's noise, as the immediacy of the phenomenon captured by the area approximation (waves, ripples, etc.) is offset by the rest of the volume analyzed. For example, the CFD noise generated by a wave is captured instantaneously and stored as an output. The same wave found in HSF does not have the same effect on the output signal since the total calculation of the liquid holdup is calculated on a broader section of the pipe, where the wave's impact has not yet happened. Despite this, a fair agreement between the two techniques is observed for the cases studied, even with differences in measurement techniques. It is noted that a maximum error of about 25% is calculated, which leads to the conclusion that the developed CFD model successfully replicated the phenomenon.

**Figure 12:**
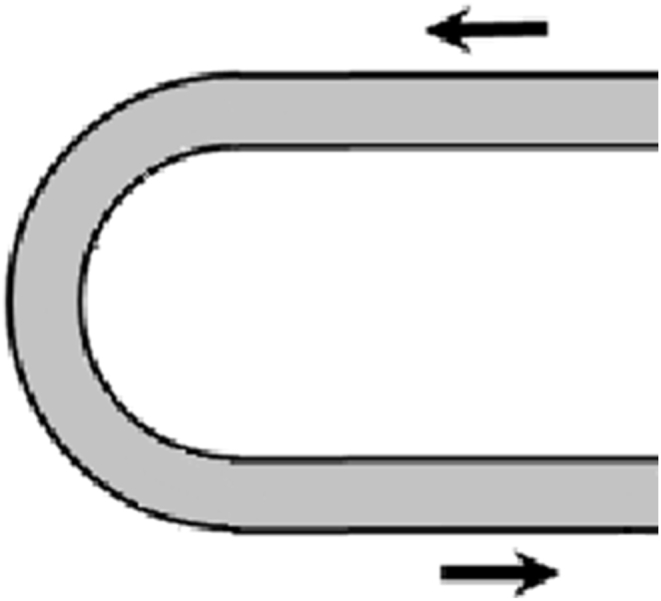
Flow direction in the figures of liquid film distribution.

Finally, a visual comparison of the distribution of the liquid film in the bend is performed. In the experimental facility, high-quality images of the mixture passing through the bend were captured using a Canon Rebel T3i Eos 600D camera, with a Canon efs 18–135 mm lens. The camera was in the inferior part of the bend facing up. The direction of the flow observed in the pictures is presented in Figure 12. In CFD, the same view as the one obtained in the facility was replicated.

**Figure 13:**
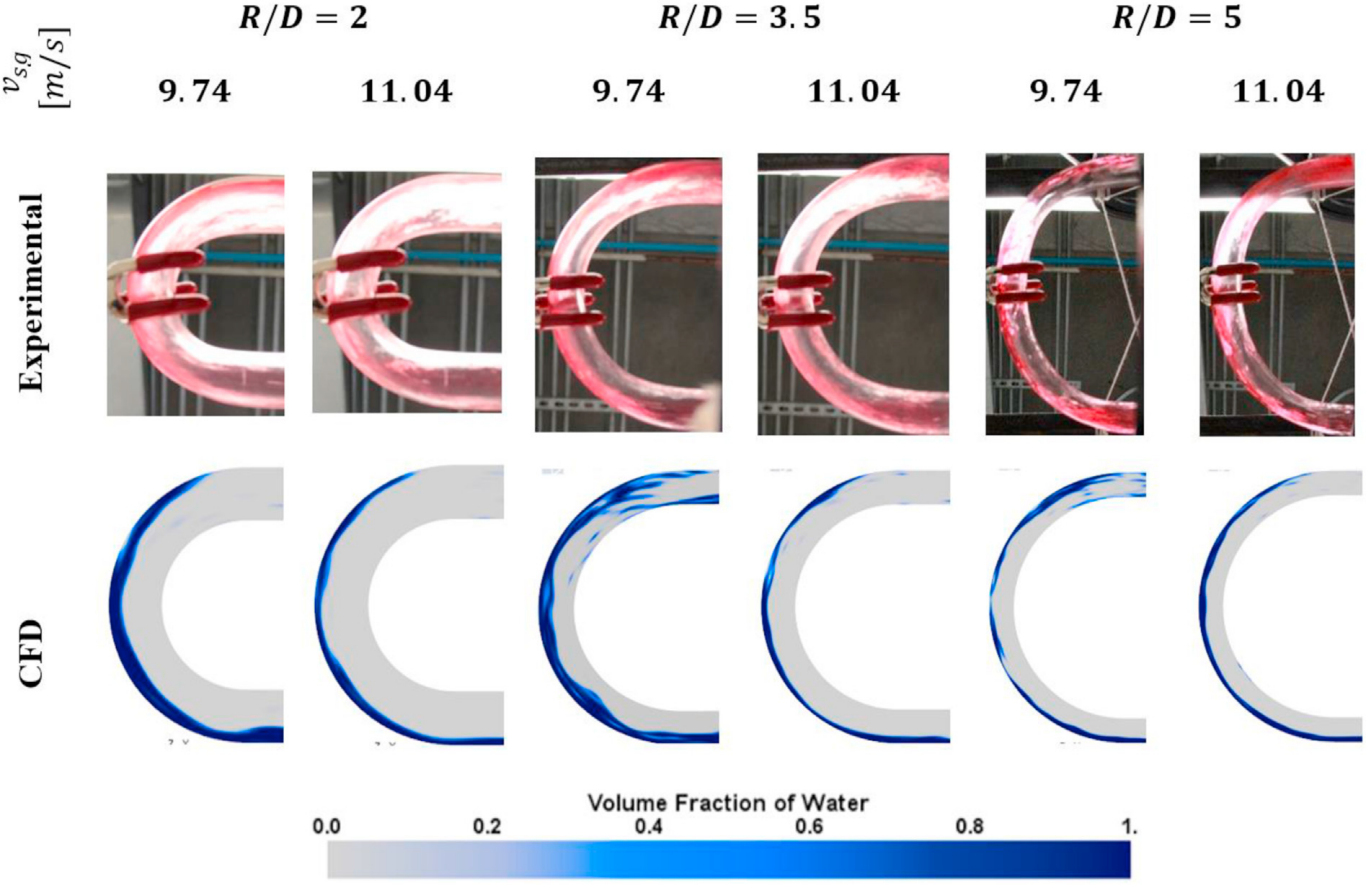
Liquid film distribution. Experiments vs. CFD.

Figure 13 presents the comparison between the results of liquid film distribution obtained experimentally and numerically.

As seen in the figure, despite the gas velocity or the bend's curvature radius, the liquid film's precise distribution cannot be determined by experimental observation. It is only possible to see that the liquid film's tendency is to be located outside the bend for all the cases. This tendency is validated with the CFD results where, for all cases, the liquid film is located on the outside of the bend.

Comparing the experimental results and the numerical results demonstrate that the CFD code correctly describes annular flow behavior through U-bends under experimentally presented conditions. Based on these, additional results were obtained from the simulations. Therefore, different variables of interest can be extracted from the numerical code. This is the liquid film distribution before and after the bend and the liquid film's velocity in the bend.

**Figure 14:**
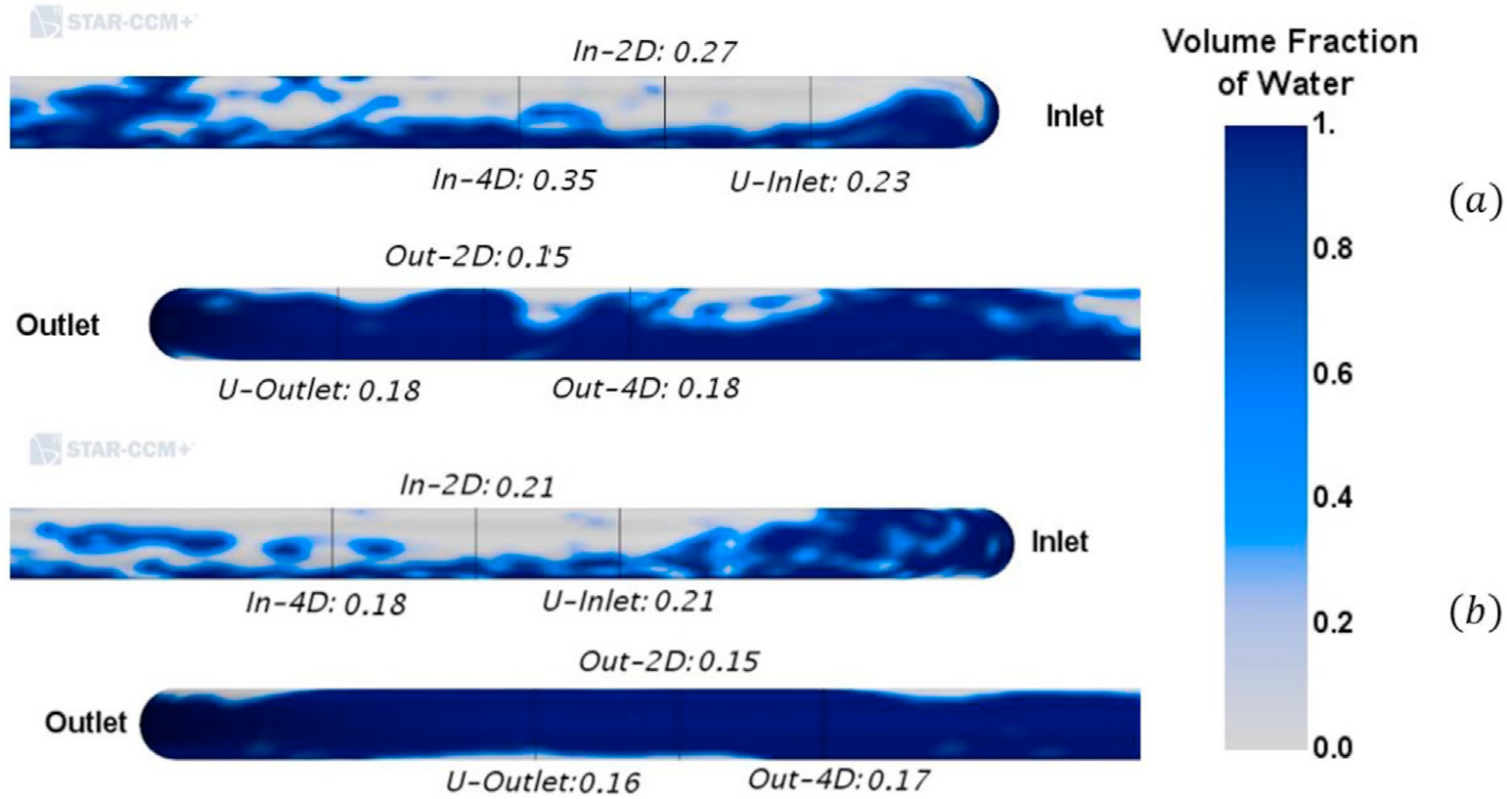
Graphical description of the liquid film before and after the bend. a. R/D = 2. v_sg = 9.74 m/s. b. R/D = 5. v_sg = 11.04 m/s.

As mentioned before, an accumulation of the liquid is observed before the bend. To further validate the observation, the liquid film's behavior upstream and downstream of the bend can be analyzed. Table 8 presents the CFD average liquid holdup (in the final 2.0 s of the simulation) before the bend and the percentage change observed after the bend. If the change is positive, a higher value of holdup was observed after the bend; if it is lower, the liquid holdup decreases after the bend. The data is extracted 4D and 2D away from the bend (both up and downstream) and at the bend's inlet and outlet. Figure 14 shows a visual description of this behavior.

**Table 8:** Liquid Holdup before and after the bend.

Case	$v_{\mathrm{sg}}\;$ [m/s]	R/D	Liquid Holdup					
			4-D		2-D		Bend	
			Before	Change [%]	Before	Change [%]	Before	Change [%]
1	9.74	2	0.35	-48.57	0.23	-44.44	0.25	-45.45
2	11.04	2	0.34	-52.94	0.35	-54.29	0.16	-48.57
3	9.74	3.5	0.21	-9.52	0.23	-13.79	0.25	-4.35
4	11.04	3.5	0.21	-14.29	0.22	-34.62	0.17	-18.18
5	9.74	5	0.19	-5.26	0.22	-15.00	0.17	-18.18
6	11.04	5	0.28	-5.56	0.21	-28.57	0.12	-23.81

The first phenomenon observed in the table is the mixture's unsteady behavior. This is because the values of holdup change as the mixture gets closer to the bend, and also, the values do not match the mean values of liquid in the visualization box (located further upstream of the bend). It also explains the accumulation of liquid observed before the bend. For all cases, the holdup after the bend (i.e., bend outlet, two and four diameters after the bend) presents a lower value than the holdup values before the bend (i.e., bend inlet, two and four diameters before the bend). This is presented as an adverse change in the holdup percentage. It is also observed that the reduction in holdup can be up to 50% in the smaller bends. This evidence the significant effect of the bend in the mixture. Figure 14 presents the water fraction of water in the outside of the pipe for both before and after the bend.

**Figure 15:**
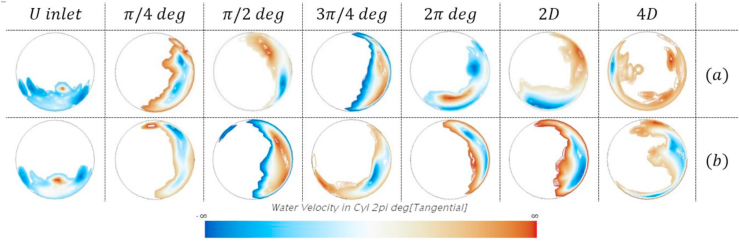
The tangential velocity of the liquid film. a. R/D = 2. v_sg = 9.74 m/s. b. R/D = 5. v_sg = 11.04 m/s.

This visual representation of the liquid film does not allow us to see the liquid film's reduction after the bend. There is more liquid for the pipe's outlet section than the inlet pipe, although the values of liquid holdup evidence the opposite. This is due to an apparent rotation of the liquid film in and after the bend. The rotation is caused by the secondary gas flow and the effect of the bend's centrifugal force. To confirm this, an approximation to the liquid velocity was obtained. Since the in VOF both phases share the velocity profile, the liquid velocity can be approximated by dividing the fluid domain into two separate sections: gas only (where the holdup is above 0.5) and liquid only (holdup below 0.5). This is to validate the observed rotation of the liquid film. In the gas-only section, the velocity is defined as 0, and in the liquid only section, the velocity profile is left unmodified. This approximation allows obtaining the velocity of the liquid in any section of the pipe or curve. An analysis of the tangential component of the velocity was made in different tube sections to validate the liquid film's rotation. Figure 15 presents the tangential velocity profile of the cases shown in Figure 14. It is worth mentioning that the liquid film's colorimetry behavior is similar for the remaining four cases. Thus, the validation of the rotation of those two cases can be extrapolated to the remaining ones.

Figure 15 presents the tangential velocity contours of the liquid in 7 different sections of the pipe, 5 of those in the bend (from the beginning, every π/4°), and 2 and 4 diameters after the bend a normal view aligned with the flow direction. If the velocity profile is brown, this means that the film is rotating counterclockwise. If the velocity profile is blue, the film is rotating clockwise, and if it is transparent or nearly transparent, the tangential velocity is near zero. As the figure intends to provide evidence of the liquid film's rotation, the magnitude of the velocity is not a critical parameter, and, as such, it was not considered.

At the bend entrance, the liquid film is located at the lower section of the pipe, and its core is not rotating as it is near transparency. The interface between gas and liquid seems to be rotating due to the secondary gas flow. However, it is interesting to observe that once the film enters the bend (i.e., π/4° and beyond), the film moves to the outside section of the bend and rotates by climbing the outlet wall of the pipe. Additionally, it is observed that once the film gets to the upper section of the pipe, it falls due to gravity and starts to rotate in the opposite direction. Once the mixture leaves the bend, the film's rotation is still observed, even four diameters after the bend. For the small curvature radius (Figure 15a) at four diameters after the bend, it seems that the film performed a full rotation around the bend as some liquid is observed on the inner section of the pipe, and it still has a positive magnitude in the tangential velocity. This shows the effect of the secondary flow and centrifugal force on the behavior of the annular flow. Future studies must be performed to examine the threshold for detecting the mixture's curvature effect; this could be accomplished by analyzing additional pipe sections downstream from the bend.

## 4 Conclusions

U-bends are standard and common accessories present in gas processing and gas transportation facilities and industrial applications. This makes their study highly relevant—especially in annular flow cases—to understand the behavior and the consequence on the return in an intricate flow pattern. This study contemplates an experimental and computational approach to the topic. The study aimed to validate and complement all the experiments with CFD simulations.

• The optical measurement technique used in this study allowed quantifying and studying a time trend of liquid holdup in straight sections of pipes, allowing researchers to identify and analyze the effect of downstream accessories in the tube such as the bends. These experimental measurements were used as a validation for the CFD results. The experimental measurements are based on a volumetric approximation to the liquid holdup, while the CFD results are based on an area approximation. Part of the observed discrepancy between the two approaches is attributed to the previously mentioned difference.
• It was possible to develop a CFD methodology that correctly predicts annular flow behavior in straight pipes and bends under the studied conditions. The method can capture, quantify, and identify crucial multiphase factors such as liquid film distribution in the bend and the straight pipe and the film's behavior and rotation induced by the bend. Useful parameters such as describing the velocity profiles in bends and the characterization of accessories were studied.
• It was possible to demonstrate the bend's effect on the mixture, especially in the liquid film's effect before the bend. An accumulation of liquid was observed upstream of the bend, especially at tighter curvatures. Also, the behavior of the film after the bend was analyzed. At a lower curvature radius, a more significant reduction in the holdup was observed after the bend, where, in some cases, a 50% reduction in the holdup value was observed. Hence, it can be concluded that the bend can work as a flow conditioner.

## Declarations

### Author contribution statement

J. López: Conceived and designed the experiments; Performed the experiments; Analyzed and interpreted the data; Contributed reagents, materials, analysis tools or data; Wrote the paper.

N. Ratkovich & E. Pereyra: Conceived and designed the experiments; Analyzed and interpreted the data; Contributed reagents, materials, analysis tools or data; Wrote the paper.

### Funding statement

This research did not receive any specific grant from funding agencies in the public, commercial, or not-for-profit sectors.

### Data availability statement

Data included in article/supp. material/referenced in article

### Declaration of interests statement

The authors declare no conflict of interest.

### Additional information

No additional information is available for this paper.
